# Current status, challenges and the way forward for dairy goat production in Asia – conference summary of dairy goats in Asia

**DOI:** 10.5713/ajas.19.0272

**Published:** 2019-07-01

**Authors:** Juan Boo Liang, Pramote Paengkoum

**Affiliations:** 1 Institute of Tropical Agriculture and Food Security, Universiti Putra Malaysia, 34300 UPM Serdang, Malaysia; 2 School of Animal Technology and Innovation, Institute of Agricultural Technology, Suranaree University of Technology, Nakhon Ratchasima, 30000, Thailand

**Keywords:** Asia, Dairy Goat, Goat Milk, Milk Price, Asian-Australasian Dairy Goat Network

## Abstract

Asia hosts more than half of the world’s 1 billion goats and is also where domestication of wild goats began. Goats, including dairy goats, are adapted to a wide variety of harsh environments and thus play key roles as providers of nutrition, food security and socio-economic status to their human owners in many low-income Asian countries. In many countries in Southeast and East Asia, medium and large scale commercial dairy goat farming can be profitable enterprises because of the high price of goat milk, and good demand due to its health and medicinal properties. In some Asian countries, dairy goats play important roles in non-commercial activities, including use as educational animals in elementary schools in Japan and show animals in Indonesia. Dairy goat farmers in Asia are faced with numerous challenges, such as a shortage of high producing animals adapted to the local environment, lack of quality feeds during a prolonged dry season, many diseases and difficulty getting their product to market, however, the increasing demand for goat milk in the newly developed and developed economies in Asia provides an optimistic future for dairy goat production in this region.

## INTRODUCTION

Asia is often called the home of the goat because the region hosts 60% of the 1 billion world goat population [1]. It has also been claimed that domestication of wild goats began in Asia (Zagros Mountains in Gangi Dareh, Iran) more than 100 centuries ago [2]. The majority of the goats in Asia, including dairy goats, are in the hands of small-scale farmers, many of them are resource-poor and landless [3]. Although goats raised under typically harsh environmental conditions in Asia continue to be important providers of nutrition, food security and socio-economic status to their human owners, they remain neglected in the overall national development policy in many countries. On the other hand, demand is surging due to a belief in its medicinal value, so goat milk is sold at a price 2 to 3 times higher than that of cow milk in many east and south-east Asian countries such as China, Japan, Malaysia, Indonesia, Thailand, and Vietnam. Large industrial and medium-scale dairy goat farms are on the increase in these countries to meet the rising demand for goat milk, but the sustainability and success rate of many of these farms are below expectations. The Asian-Australasian Dairy Goat Network (AADGN), established in 2012, provides a platform for producers and researchers to exchange experience and networking to promote and support this challenging industry. The success of AADGN is evident by the increasing number of participants over the last four conferences organized by member countries of the Network. The major technical constraints identified by AADGN member countries in developing dairy goat farms were supply and quality of breeding goats, feed resources, health, parasite management, marketing through strong producer organisation and technology application [3].

This paper reviews and summarises presentations from the 4th Asian-Australasian Dairy Goat Conference (AADGC2018) held in September 2018 in Tra Vinh, Vietnam. It covers issues highlighted by the speakers related to the challenges and strategies to bring forward the dairy goat industry in Asia and beyond. The full Conference Proceedings are available at: https://aadgc2018.tvu.edu.vn

## HIGHLIGHTS OF PAPERS PRESENTED

### Plenary papers

Seven plenary papers covering diverse areas relevant to dairy goats were presented. They include an overview of the global dairy goat production, progress in feeding and nutrition, reproductive physiology and breeding, and potential use of dairy goats in adapting to and mitigating climate change. In addition, a video was shown entitled “Peeking into the goat milk business” demonstrating how goat farmers in a village in Indonesia turned small-scale dairy production into a viable and innovative business. The link to the video is: https://www.youtube.com/watch?v=0Yp8uLvrYXw&feature=youtu.be

All together, these presentations provided information on recent advances that are useful for farmers and producers to adopt to improve goat milk production on small farms or in industrial situations.

#### Global dairy status and development

**Figure 1:**
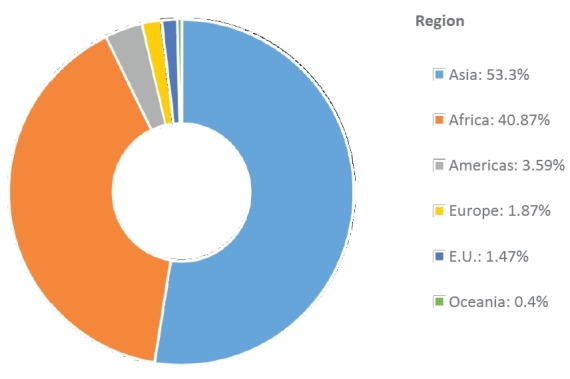
Goat population in the different regions (% of world population) in 2017.

The president of the International Goat Association provided a comprehensive summary of the global status of goats, with special emphasis on Europe and the United State [4]. Although the percentage rise in the goat population over the last decade (2000 to 2013) was highest in Oceania (65.8%), followed by Africa (48.6%) and then Asia (30.2%), Asia remained as the top producer in the world—more than half of the world’s 1 billion goats are in Asia (Figure 1). The author highlighted that there are lessons that goat milk industries in Asia can learn from their counterparts in Europe, particularly France, to enhance development of their dairy goat industries. The areas include governmental support, marketing systems, responding to changing consumer demand, using new technology, and implementing policies for beneficial social and environmental impacts.

#### Feeding and nutrition of dairy goats

Fifty-two percent of the world’s 202 million dairy goats are found in Asian countries, primarily China, India, Bangladesh, Pakistan, Indonesia and Afghanistan. However, the average milk yield per head is only a quarter (26%) of that for Europe (762 vs 2,901 g/d) [5]. Feeding and nutrition are key factors influencing the productivity and milk yield of dairy goats. To minimize cost, grazing is the main production system for goats in developing countries [6]. However, quality and quantity of forage in open grazing systems are subjected to seasonal fluctuations and in many tropical Asian countries, high-quality roughages are often in short supply during long dry and hot seasons. Agro-industrial byproducts are often used as feed supplements during these difficult periods. Because of their tolerance to many plant secondary metabolites, dairy goats can consume a wider variety of byproducts and non-conventional feedstuffs compared to other livestock, under different production systems [7]. Recently, a variety of byproducts containing natural antioxidants are receiving increasing attention from researchers, including those in Asia, in the feeding of dairy goats. These include polyphenol-rich pineapple rind meal [8] and anthocyanin-rich purple corn stover silage [9]. The increasing interest in using natural antioxidant-rich byproducts in dairy goats stem from i) benefits to the host animal because dairy goats experience high stress from the metabolic demands for maintenance and milk production relative to other small ruminants [10], and ii) these antioxidant compounds were reported to be capable of being absorbed and deposited into the milk [9, 11]. This latter aspect should provide an additional marketing advantage as “natural antioxidant-rich” goat milk.

Proper feeding and nutrition is a key to high fertility in female dairy goats as well as to allow expression of their genetic potential for milk production. It is well known that dairy goats in Asia (and also other regions) are managed under a wide variety of production systems, ranging from small herds grazing public land to mid-size and large scale industrial farms. Thus, feed resources available for dairy goats vary not only from farm to farm but also among seasons – particularly on small farms. Goetsch [7] provided a comprehensive overview of recent research of feeding practices and nutrition of lactating dairy goats. Using the study of Eknaes and coworkers [12], the author noted that lactating dairy goats can mobilize a large portion of their body fat to support milk production under different production conditions; however, management thereafter should facilitate recovery thereafter.

#### Research progress on reproductive physiology and breeding technology of dairy goats

Luo and Sun [13] in their presentation highlighted that currently, there are more than 50 well-known European breeds of dairy goats used as genetic resources by many countries in Asia as purebreds and also for crossbreeding to develop new breeds adapted to local conditions. Although many countries, including China, have made great advances in feeding management, estrus control, artificial insemination and embryo transfer to support their national dairy goat breeding program, control of reproductive physiology remains challenging on the farm. For example, PMSG or FSH is an effective protocol for estrus synchronization in dairy goats [14], but repeated superovulation can harm the ovary and thus limit reproductive performance [15]. However, the authors were optimistic that appropriate technologies will be developed to overcome the above setback.

#### Goat production as a strategy for sustainable ruminant production in the face of climate change

Climate change is a worldwide phenomenon that encompasses increased global temperature, more distinct and prolonged droughts and flooding, and depletion of natural resources [16]. Based on the Global Climate Risk Index compiled by Germanwatch, Vietnam, Myanmar, the Philippines and Thailand, in Southeast Asia, are among the 10 countries in the world most affected by climate change in the past two decades [17]. If left unchecked, the economic impact of global warming on the above countries and other Southeast Asian countries in general, could be devastating with an estimated 11% reduction in GDP by the end of the century - primarily in the agriculture, fishing and tourism sectors. Several countries in this region, such as Vietnam, Indonesia and the Philippines have long coastlines, so rising sea levels together with prolonged dry seasons will cause increased groundwater and soil salinity that will be very detrimental to agriculture and livestock production, according to two presentations relating to goat production and climate change [18,19]. Rising seawater in low lying coastal areas increases soil salinity and changes plant populations, resulting in relatively more browse-type plants that goats consume, and fewer grasses that cattle and sheep prefer. Also, goats appear able to consume water higher in salinity than sheep and cattle and, thus, presumably would be able to perform well despite consumption of plants high in minerals (halophytes). Relatedly, the ability or preference of goats to consume a wide array of plants if available probably gives them an advantage compared with cattle and to a lesser extent sheep that tend to consume a fewer number of plant species even when many different ones are available.

In recent years, livestock and in particular ruminant farming has been viewed negatively in the eyes of the general public because of their contribution to greenhouse gases (GHG). Although in terms of global methane production, goats contribute less than 10% of that contributed by cattle and other larger ruminants, mitigating methane production from goats can meaningfully contribute to the overall mitigation of global GHG. Strategies including integrating goats in agroforestry systems to browse leaves from trees and converting the residual branches and stems via gastification to a combustible gas and biochar, the latter acting as the vehicle to sequester carbon to the soil [19]. Other methane mitigation research conducted recently includes a comparison of extensive farming vs intensive farming system [20], replacing cereal grains with fibrous byproducts [21], supplementation of coconut oil [22], and supplementation of naturally-produced lovastatin from palm kernel cake [23].

### Reports by AADGN member countries

A total of eight Country Reports from “member countries” of the AADGN were presented in the AADGC2018 conference: three from South-Asia (Iran, India, and Pakistan), two from East-Asia (Japan and Taiwan), three from Southeast Asia (Indonesia, Philippines, and Vietnam) and one from Australia. Below is the summary of the above presentations with some additional information from the Country Reports 2013/14 submitted to AADGN and the 3rd AADGC conference (AADGC2016) held in Yangling, China in 2016.

#### South Asia

##### i) Iran

**Figure 2:**
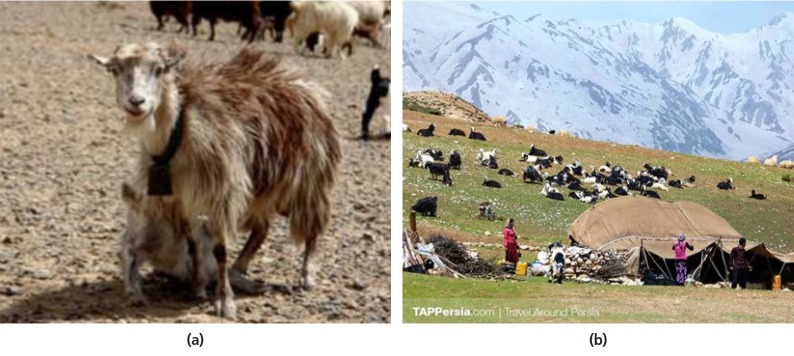
(a) A typical Nadoshini goats (*courtesy of Mr. Seyed Mehdi Hoseini*); (b) Nomadic goat farming in western Iran (*courtesy of Mr. Seyed Mehdi Hoseini*).

Hosseini and Kalantar [2] reported that the current goat population in Iran is approximately 20 million head, supplying about 12% of the country’s total meat production (88,000 tons) and 4% of the country’s raw milk (304,000 tons). In addition, goats in Iran are an important source for its famous hair and mohair industry which directly or indirectly employs up to 1.2 million people in the country. Approximately 90% of the goats in Iran are located in the dry regions of the country. Examples include the Nadoshani goat (Figure 2a), which produces high value milk and cashmere in the driest areas (less than 100 mm annual rainfall) in central Iran. Over the centuries, goats played a vital role in many rural communities in Iran, including the traditional nomadic tribal regions (Figure 2b), and it has been claimed that domestication of wild goats was started in the Zagros Mountains in Gangi Dareh (Iran) around 10,000 B.C. Realizing the important of goats, there is strong governmental support for developing the goat industry in Iran, aiming to double the milk production from goats in the next 10 years. To achieve the above target, the governmental programs include preservation of native goat breeds while upgrading their productivity through crossbreeding with imported pure bred dairy goats, especially from Europe.

##### ii) India

Globally, India has the largest population of goats and is also the top producer of goat milk (5.75 million MT annually in 2016–2017), making up about 25% of the global goat milk produced annually. In term of national milk production, goat milk made up 3.5% of the milk produced, while the remainder comes primarily from cows and buffaloes [24]. Approximately 83.4% of goats in India belong to landless small-scale farmers in ecologically vulnerable and drought prone areas. More sustainable farming systems including goat rearing could improve for the communities in harsh environments in rural India, but will require political will, financial resources, high quality technical training and animal care, and low cost to send products to markets. Some commercial scale dairy goat farms have developed to take advantage of the increasing demand and high price for goat milk, due to appreciation of its medicinal and health properties in some niche markets. Some goat milk is processed into cheese as well. However, growth of the sector is relatively slow.

**Figure 3:**
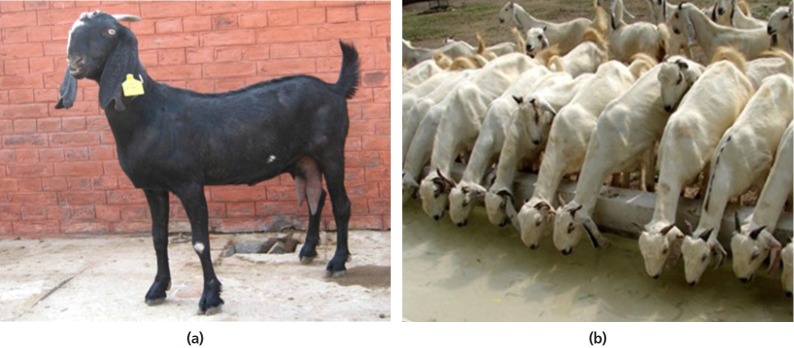
(a) Beetal doe (*courtesy of Dr A. K. Thiruvenkadan*), (b) A herd of Jamunapari goats (*courtesy of Dr A. K. Thiruvenkadan*).

Processing goat milk into high-value products particularly goat cheese, is a good way to expand dairy goat farming in India. Nearly INR156 million worth of goat cheese is imported annually for the local premium hotels and restaurants. Indian goat cheese could possibly compete in the international market as well. In order to achieve long-term success, the French approach which focuses on governmental support, improving marketing systems, responding to changing consumer demand and using more technology [4] provides a model to follow. In addition, India is comparatively rich in genetic resource with 28 goat breeds of which 4 are milk breeds (e.g. Beetal, Figure 3a) and 8 dual-purpose breeds (e.g. Jamunapari, Figure 3b) [25]. Together with the on-going genetic improvement programs supported by the government provide the path to successful expansion of the dairy goat industry in India.

##### iii) Pakistan

**Figure 4:**
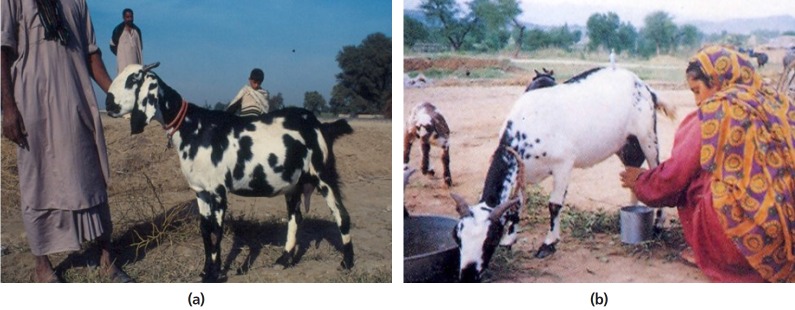
(a) A typical female Beetal goat in Pakistan (*courtesy of Dr. Fatah Ullah Khan*); (b) Woman milking her goats in Punjab (*courtesy of Dr. Fatah Ullah Khan*).

Similar to several low-income neighboring countries, goats play important socio-economic roles in the livelihood of rural farmers in Pakistan; providing them with meat, milk, fibre and skins. About 6.8 million farmers are involved in raising the 74.1 million goats in Pakistan. Traditionally, small ruminants such as goats and sheep are raised under four main production system; namely nomadic, transhumant, household and sedentary in various regions in Pakistan. However, due to degradation of rangelands, and increasing drought and flood, the production system of the small ruminants, especially dairy goats, have shifted from an extensive grazing system to the confined and household systems where women play a key role in managing the animals (Figure 4a and 3b) [26].

#### East Asia

##### i) China

**Figure 5:**
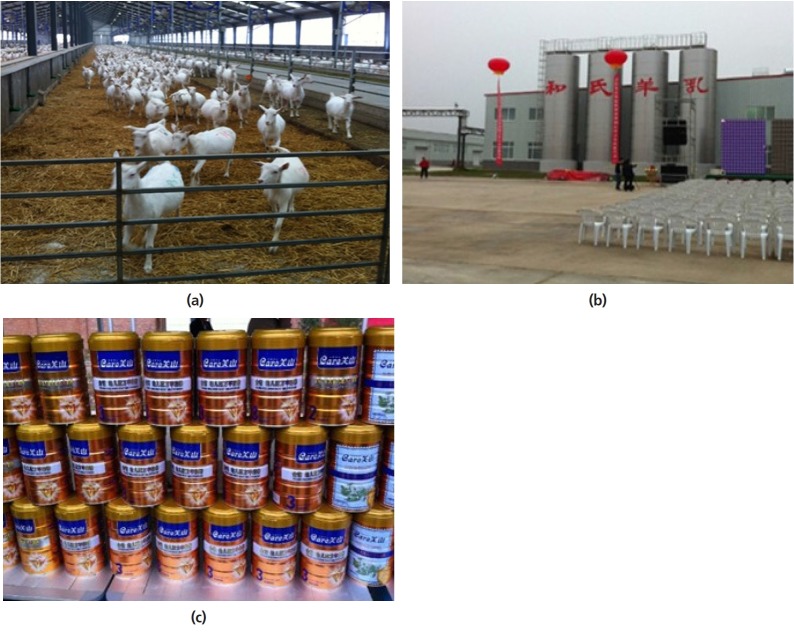
One of the newly established dairy goat operations in Yangling, China which combines (a) production, (b) processing and (c) marketing of its products.

Goats in China (183 million head) constitute 18% of the total world goat population. The total number of dairy goats in China currently stands at 12 million distributed over the 28 provinces of China with nearly half concentrated in Shaanxi province (2.3 million), Shandong province (2.1 million), Henan province (1.17 million), Hebei province (0.67 million), Gansu province (0.56 million), and Xinjiang province (0.53 million). Historically, there was no specialized dairy goat breed in China. Existing good dairy goats are the result of crossing local and exotic dairy goats from Europe during the late 19th and early 20th centuries. Many breeds of dairy goats have been officially recognized in China, but the main ones are Xinong Saanen, Guanzhong, Laoshan, Nubian and Henan [27]. Due to the rapid increase in demand for goat milk, large scale commercial goat milk enterprises have been established in the last few years in China (Figure 5). They combine production, processing and marketing their own products.

##### ii) Japan

**Figure 6:**
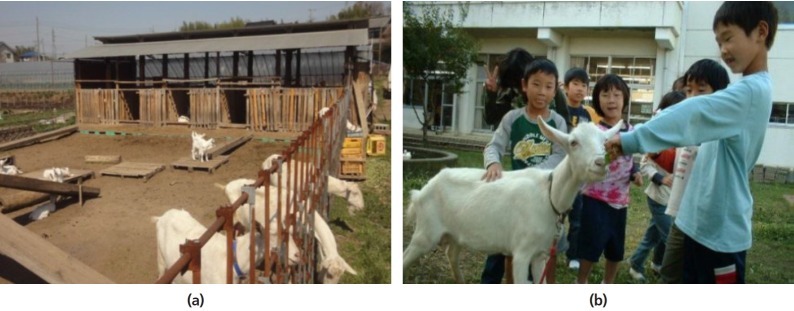
(a) A typical goat farm in Japan (*courtesy of Dr. Shinichi Kobayashi*); (b) Goats for educational purposes in Japan (*courtesy of Mr. Akio Imai*).

The development of the dairy goat industry in Japan is rather unique and is greatly influenced by the pace of economic growth over the last half century. In the 1950s goats were kept to occasionally supply meat and milk for the family and for controlling noxious weeds around rice fields [28]. However, that role decreased, and resulted in a decline in population from approximately 700 thousand in 1957 to 20 thousand in 2015 as the Japanese economy grew rapidly during the same period of time. There was a slow increase in the goat population over the last few years due to increasing interest in the use of goats for non-commercial activities, such as control of noxious weeds, as educational animals in elementary schools (Figure 6) and companion and hobby animals [29].

There is no formal marketing system for goat milk and goat milk products in Japan. These items are sold by approximately 20 individual goat farms at lucrative prices. Challenges faced by the dairy goat industry in Japan include (i) insufficient number of A.I. technicians to support the breeding program at farm level, (ii) meeting the minimum standard of milk quality for sale (3.6% and 8.0%, respectively, for milk fat and non-fat solids) because most small farms allow their goats to graze with only some feed supplements, and (iii) occasional occurrence of diseases such as CAE (*Caprine arthritis-encephalomyelitis*) [28].

##### iii) Taiwan

**Figure 7:**
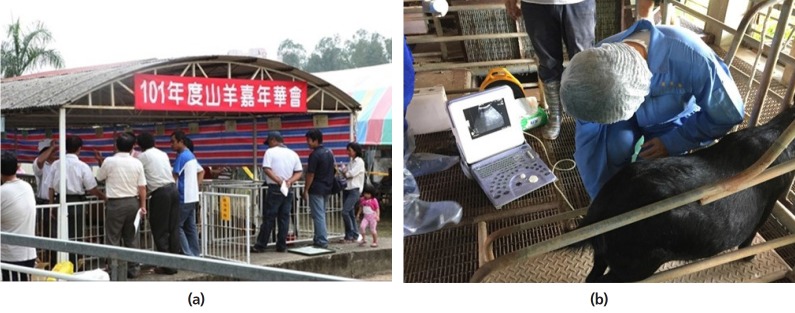
Dairy goat (a) conference and (b) workshop to transfer technologies to farmers and industry (*courtesy of Dr. Hsia Liang Chou*).

Approximately 94% of the total 45,365 dairy goats in Taiwan are located in south Taiwan. The dairy goat industry is comparatively well-developed. There are 260 specialized dairy goat farms with an average size of 105 milking goats (mainly Alpine, Nubian and Saanen) producing an average 2.5 to 3.5 kg/d. Dairy goats are kept under an intensive system with specialized companies marketing fresh goat milk in different flavors and products such as goat milk yogurt, calcium tablets, soap, and shampoo. In term of technical support, a total of 6 national and regional universities and research institutions are involved in various disciplines of dairy goat research. Conferences and workshops (Figure 7) are regularly held to transfer research technologies to the farmers and the industry [30].

#### Southeast Asia

##### i) Indonesia

**Figure 8:**
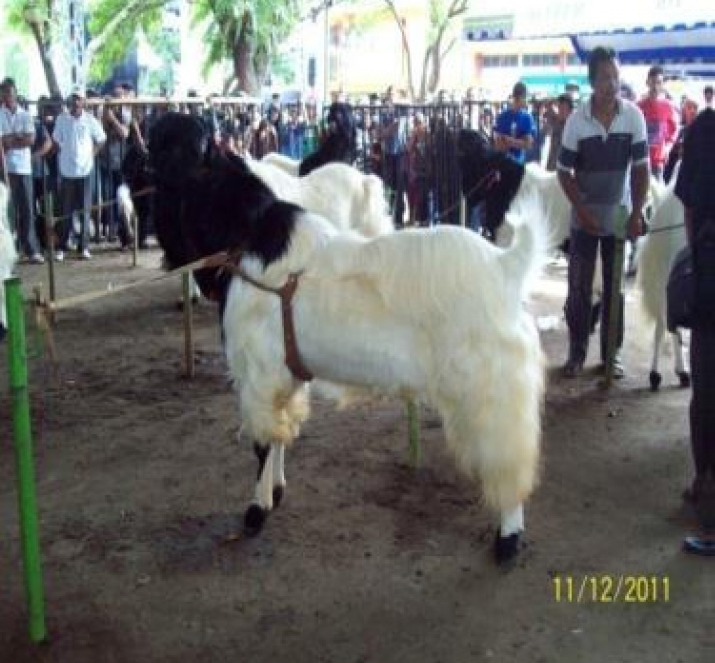
An annual event on Etawah goat contest in Central Jaya (*courtesy of Dr. Dewi Astuti*).

The population of goats in Indonesia has increased swiftly, from 14.5 million in 2007 to 17.5 million in 2011. The goats are spread throughout the 33 provinces with the highest population in Central Java (21%), followed by East Java (16%), and West Jaya (11%). Out of the total population, approximately 4 to 5 million (32%) are kept for milk production [31]. Several national and regional efforts have been implemented in the past to promote drinking of goat milk to increase local demand but have achieved only limited success. To ensure better success, the government, working with the Indonesia Goat and Sheep Association (Himpunan Peternakan Domba dan Kambing Indonesia), is implementing a national sheep and goat development program in Indonesia. The dairy goat portion focuses on (i) breeding and selection through culture-based contests and standardization of the locally prominent Peranakan Etawa breed (Figure 8), (ii) improving the local breeds through crossbreeding with imported breeds to enhance milk production and quality, and (iii) the development of a dairy goat Cluster Farming Community program based on the on-going successful meat goat model in the country [32].

##### ii) Malaysia

**Figure 9:**
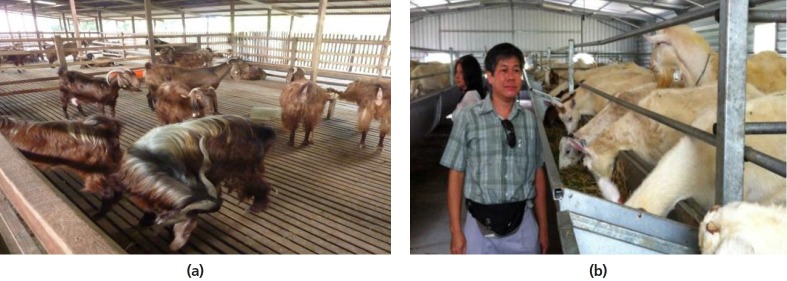
(a) Shami goats raised on slatted floor in Malaysia (*courtesy of Dr. S. Shanmugavelu*); (b) Saanen goats raised in a closed house with evaporative cooling in Malaysia (*courtesy of Mr. Ivan Ho*).

Although dairy goat farming has been in existence for more than fifty years in Malaysia, there was a recent resurgence of dairy goat production attributed to the increasing demand for goat milk primarily because of the traditional belief of its health benefits. The main dairy goat breeds in Malaysia are Saanen, Anglo Nubian, and Jamnapari. More recently, dual-purpose Shami goats (Figure 9a) were introduced. Almost all commercial dairy goats are kept in raised slatted floored housing to prevent intestinal parasites, which is one of the major problems faced by producers in Malaysia. Cut grass and legume forages, commercial feed concentrates, and on some farms, agro-byproducts such as palm kernel cake and oil palm fronds are fed to the animals. The main driving force for the recent resurgence in dairy goat production in Malaysia is the high price of goat milk which on average fetches US$ 3.75 per liter (ex-farm price) as compared to US$ 0.70 per liter for cow milk. Goat milk is generally sold fresh directly by farmers to individual and regular customers although some are processed and then marketed in the supermarkets in local cities and Singapore [33]. A local dairy goat farm that adopted the ventilated closed house system has shown that high productivity of imported pure bred goats can be achieved under the hot and humid environments in Malaysia (Figure 9b).

##### iii) Philippines

Alo et al [34] reported that the dairy goat industry in the Philippines is a sunrise industry, with a 14% growth in 2017. Although the dairy goat population stands at about 14 thousand head making up less than 0.4% of the total goat inventory of 3.72 million in 2017, it is expected to grow under the on-going government initiatives. Dominating the industry are about 50 commercial farms primarily located in Luzon, Mindanao, and Visayas provinces. A recent study, funded by the Philippine Council for Agriculture, Aquatic and Natural Resources Research and Development of the Department of Science and Technology (DOST-PCAARRD) reported that 95% of the dairy goat farms are commercial operations with an average of 179 does per farm. The Anglo-Nubian is the most common breed, producing about 1.2 liters per day over an average lactation period of 172 days. Most dairy goats in the Philippines are managed under confinement and fed with cut grass and legume forages, and concentrate supplement. Research is actively conducted by R&D institutes and has resulted in the commercial production of a *Indigofera* leaf meal based pelletized total mixed ration and a natural anthelmintic (*MCM Herbal Dewormer*) based on extracts of *Mimosa pudica*, *Chrysophyllumcainito*, and Tinoporarumphii which contain anthraquinone and flavonoids. Goat milk is sold for twice the price of cow milk (US$2.40–3.00 vs 1.50–1.70 per liter) in the Philippines. This, together with the government initiative is aimed to increase goat milk production by 150% by 2020 [34].

##### iv) Thailand

**Figure 10:**
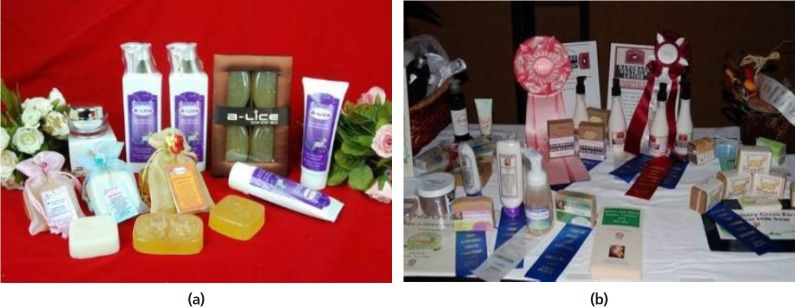
(a) Soap and shampoo products from goat milk (*courtesy of Dr Sangsak Nakavisut*); (b) Cosmetic products from goat milk (*courtesy of Dr Sangsak Nakavisut*).

Thailand has about 20,000 dairy goats, approximately 4.5% of the total goal population (440,000 head), and which are mainly located in central (near to Bangkok) and southern Thailand, where a high proportion of the Muslim population live. The Sannen is the most popular imported dairy goat breed followed by the Alpine, Toggenburg and dual-purpose Shami—many of which are used to improve the local goats through crossbreeding programs. The milk yield of the crossbred goats averaged between 0.79 to 1.38 kg/d but some farms achieving as high as 2 to 3 kg/d over a lactation period of 200 days [35]. As in the neighboring countries, the high price of goat milk is the driving force behind the expansion of commercial dairy goat production in Thailand. In addition to fresh goat milk, many products including soap, shampoo and cosmetic products made from goat milk are sold in Thailand (Figure 10).

##### v) Vietnam

**Figure 11:**
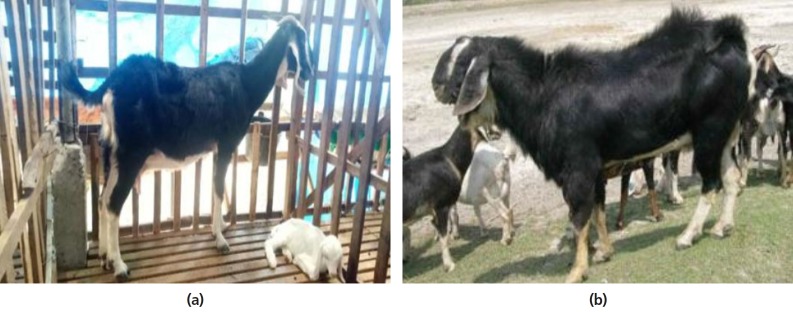
(a) Bach Thao doe with a Saanen x Bach Thao kid (*courtesy of Dr. Do Thi Thanh Van*); (b) A typical Bach Thao Buck (*courtesy of Dr Nguyen Van Thu*).

Vietnam’s total goat population increased 30.5% annually from 1.8 million in 2015 to 2.6 million in 2017. A similar rate of increase was also estimated for dairy goats (which made up about 5% of the total goat population), from 89,000 in 2015 to 128,000 in 2017 [36]. Although pure breeds such as Saanen, Alpine and Anglo-Nubian were imported to improve the local genetic resources, Bach Thao, a local dual-purpose goat, remains a popular breed among local farmers in Vietnam. The breed is believed to be a cross of Alpine, Anglo-Nubian and local breeds. The mature weight of male Bach Thao goats is comparable to that of Saanens (50–70 vs 70–78 kg) and the averaged milk yield of female Bach Thao was 1.23, compared to 2.0 kg/d for Saanens raised in Vietnam (Figure 11). The dairy goat industry is expected to continue to grow along with the national economy in the next decades.

## SYNTHESIS

More than half of the world’s 1 billion goats are found in Asia. Goats, including dairy goats, play vital role in providing nutrition, food security and socio-economic status to their owners in many low-income Asian countries. In the developing and developed countries of Southeast and East Asia, medium and large scale commercial goat farming can be profitable enterprises because of the high price of goat milk due to demand for its health and medicinal properties. Dairy goat producers in Asia can learn from their counterparts in the industrial countries such as France and Greece to improve their goat milk products and widen their markets. Dairy goat farmers in Asia faced with numerous challenges, including lack of good genetic breeding animals adapted to the local environment, shortage of quality feed, frequent occurrence of diseases and marketing. However, the increasing demand for goat milk which resulted in new ventures into the dairy goat farming in Asia provide a bright future for dairy goat production in this region. The AADGN plays a critical role in providing a platform for sharing information and networking among different dairy goat stakeholder groups to bring forward the dairy goat industry in Asia and beyond to a greater height.
